# JNSViewer—A JavaScript-based Nucleotide Sequence Viewer for DNA/RNA secondary structures

**DOI:** 10.1371/journal.pone.0179040

**Published:** 2017-06-05

**Authors:** Jieming Shi, Xi Li, Min Dong, Mitchell Graham, Nehul Yadav, Chun Liang

**Affiliations:** 1 Department of Biology, Miami University, Oxford, Ohio, United States of America; 2 College of Information Science and Engineering, Guangxi University for Nationalities, Nanning, Guangxi, China; 3 Department of Automation, Xiamen University, Fujian, China; Harbin Institute of Technology Shenzhen Graduate School, CHINA

## Abstract

Many tools are available for visualizing RNA or DNA secondary structures, but there is scarce implementation in JavaScript that provides seamless integration with the increasingly popular web computational platforms. We have developed JNSViewer, a highly interactive web service, which is bundled with several popular tools for DNA/RNA secondary structure prediction and can provide precise and interactive correspondence among nucleotides, dot-bracket data, secondary structure graphs, and genic annotations. In JNSViewer, users can perform RNA secondary structure predictions with different programs and settings, add customized genic annotations in GFF format to structure graphs, search for specific linear motifs, and extract relevant structure graphs of sub-sequences. JNSViewer also allows users to choose a transcript or specific segment of *Arabidopsis thaliana* genome sequences and predict the corresponding secondary structure. Popular genome browsers (i.e., JBrowse and BrowserGenome) were integrated into JNSViewer to provide powerful visualizations of chromosomal locations, genic annotations, and secondary structures. In addition, we used StructureFold with default settings to predict some RNA structures for *Arabidopsis* by incorporating *in vivo* high-throughput RNA structure profiling data and stored the results in our web server, which might be a useful resource for RNA secondary structure studies in plants. JNSViewer is available at http://bioinfolab.miamioh.edu/jnsviewer/index.html.

## Introduction

RNA is an informational molecule that can fold into complicated shapes. The pairing of local RNA nucleotides can create secondary structures, such as hairpins and stem–loops, and interactions among distantly located nucleotides can create tertiary structures [1]. RNA structures can influence transcription, splicing, cellular localization, and translation, and play a critical role in maturation, function, and regulation of various RNAs [1–4]. Protein-coding RNAs have some interesting secondary structure features. In *Arabidopsis thaliana*, recently, the first *in vivo* RNA secondary structure map on a genome scale demonstrated that RNA secondary structures play an important role in alternative splicing and alternative polyadenylation, as well as translational regulation [4]. Some non-coding RNAs (ncRNAs) also have special structures and functions. For example, precursor microRNA (pre-miRNA) has the hairpin structure, and the exact locations of a mature miRNA and the counterpart star miRNA along the hairpin structure of its pre-miRNA precursor are critical for validating miRNA candidates revealed by small RNA-Seq [5]. Some long non-coding RNAs (lncRNAs) have complicated secondary or tertiary structures, which are important for mediating interactions with proteins and other nucleic acids [6]. Evidence has revealed that some lncRNAs can serve as miRNA sponges and inhibit the binding of miRNAs to their target mRNAs [7,8]. More interestingly, in plants, certain RNA structures can form “RNA thermometers”, which are temperature-sensitive non-coding RNA molecules that regulate gene expression [9,10] and inhibit translation [11–13]. RNA secondary structures in plants are uniquely suitable for rapidly sensing the environmental stimuli, however, the landscape and functions of plant RNA secondary structures are not well studied [14].

In addition to RNA, single-stranded DNA (ssDNA) also has important biological functions related to its secondary structures. In DNA replication, recombination, repair, and transcription, ssDNA has to be in a proper conformation, which either allows or blocks the binding of proteins or other nucleic acids [15]. In some cases, the folded ssDNA displays enzymatic activities *in vitro*, such as cleavage and ligation of nucleic acids [16–19]. The structures of ssDNA and their functions in transcription need further research, but the available data is very limited. Now some bioinformatics software tools such as RNAstructure [20] and Mfold [21] are available for ssDNA secondary structure prediction, and can be utilized to explore the connection between ssDNA secondary structures and various biological processes.

In order to appreciate the role of DNA/RNA secondary structures in many biological processes, there is a growing demand in annotating functional sites (e.g., poly(A) sites and 3’/5’ splice sites), sequence motifs (e.g., AAUAAA poly(A) signal), and sequence fragments (e.g., introns, exons, or their boundaries) within DNA/RNA secondary structure graphs. Biologists need software that can help them appreciate the direct connection among linear sequence motifs, genic annotations, secondary structure graphs, and pairing/folding information frequently presented in Dot Bracket Notation (DBN) or Connectivity Table (CT) format. Many bioinformatics tools, such as RNA Movies [22], RnaViz 2 [23], PseudoViewer3 [24], VARNA [25] and RNAfdl [26], have been developed for visualizing RNA secondary structures. Recently, new web services such as Pse-in-One [27] and repRNA [28] have been developed to visualize the feature vectors of DNA/RNA sequences, which can be combined with machine-learning algorithms to develop computational predictors and analysis methods in bioinformatics. However, to our best knowledge, there is no implementation of DNA/RNA secondary structure viewer in JavaScript that provides seamless integration with the increasingly popular web computational environments.

To understand the interaction between RNA secondary structures and transcriptome, high-throughput sequencing technology has been utilized recently to probe genome-wide RNA secondary structures *in vivo* for *Arabidopsis* [4]. A technique called structure-seq [4] uses dimethyl sulfate (DMS) as the RNA structure probing reagents. DMS can methylate As and Cs in single-stranded regions of RNA. After DMS methylation, reverse transcription of the RNA sequences is performed, and reverse transcriptase (RT) stops one base before the DMS methylation site. After ssDNA ligation, PCR amplification, and deep sequencing, the RT stop sites can be detected with bioinformatics tools, and the single-stranded regions of RNA can be derived. Thus, structure-seq can provide experimental restraints about the single- and double-stranded regions, overcome the limitations in pure computational RNA structure prediction methods (*i*.*e*., unclear free energy parameters, RNA interaction with other molecules, and dynamical RNA folding) [29], and improve the accuracy of RNA structure prediction in certain experimental conditions. Such high-throughput RNA structure profiling data can be analyzed now by StructureFold [29], a tool that uses RNAfold [30] and RNAstructure [20] as the core prediction algorithms, restrains secondary structure predictions with *in vivo* structural data, and conducts genome-wide RNA structure prediction and reconstruction. Visualizations of predicted RNA structures from *in vivo* structural data are indispensable for researchers to explore DNA/RNA secondary structures and relevant biological functions in plants.

Here, we developed JavaScript-based Nucleotide Sequence Secondary Structure Viewer (JNSViewer) web service for DNA/RNA secondary structure visualization. JNSViewer is bundled with several popular RNA or DNA prediction software for comparative secondary structure predictions, including RNAfold [30], RNAshapes [31,32], RNAsubopt [30], RNAstructure [20] and Mfold [21], and users can customize RNA structure predictions with different programs and settings. JNSViewer can provide precise and interactive correspondence among nucleotides, pairing/folding data in dot-bracket format, secondary structure graphics, and genic annotations. Users can add customized genic annotations in GFF format to structure graphs, search for specific linear DNA/RNA motifs, and extract the structure graphs of sub-sequences. JNSViewer allows users to choose a transcript or specific segment of the *Arabidopsis* genome sequences and predict the corresponding secondary structure. We integrated popular genome browsers: JBrowse (http://jbrowse.org/) [33] and BrowserGenome (http://www.browsergenome.org/) [34], and created individual transcript tracks for 8 different categories of RNAs (i.e., mRNAs, miRNA, tRNA, rRNA, snRNA, snoRNA, transposon elements, and other ncRNAs), providing powerful search, filtration, and visualization of chromosomal location, gene structural annotation and relevant RNA secondary structures. In addition, we used StructureFold with default settings to predict some RNA secondary structures for *Arabidopsis* including protein-coding and non-coding RNAs by incorporating *in vivo* high-throughput RNA structure profiling data [4] and stored the results in our web server, which could be a useful resource for RNA secondary structure and function studies in plants. We also stored the results of *in silico* RNA structure prediction for *Arabidopsis* by RNAfold with default settings and users can easily compare them with the *in vivo* structures.

## Results

### Overall design

The web interface of JNSViewer is implemented with HTML, CSS, and JavaScript. The server-side code is written in C++, PHP, and Perl, and the backend database is essentially a flat-file database residing in Ubuntu 14.04 LTS system. The design of software and data processing pipeline are described in **Methods**. JNSViewer provides three major functions: (1) DNA/RNA secondary structure prediction with bundled software; (2) secondary structure graph visualization using input file that contains structural pairing information in DBN, CT or SSDJ format; (3) interactive visualization of chromosomal locations, genic annotations, sequence motifs and secondary structure graphs by integration of popular genome browsers (i.e., JBrowse [33] and BrowserGenome [34]). In general, users have 4 different ways to use our web server: (1) upload a single query DNA/RNA sequence in FASTA format with an optional genic annotation GFF file, and customize the prediction algorithm; (2) upload a file that contains structural pairing information in DBN or CT format with an optional genic annotation GFF file; (3) search for a transcript of *Arabidopsis*, predict (optional), and visualize its RNA secondary structure; (4) specify any sequence fragment of the *Arabidopsis* genome for secondary structure prediction of ssDNA or its putatively transcribed RNA. Eventually, users can view or download secondary structure graphs in PNG or SVG format.

In particular, JNSViewer has the following important features: (1) Comparative DNA/RNA secondary structure prediction with 5 popular prediction tools (i.e., RNAfold, RNAshapes, RNAsubopt, RNAstructure, and Mfold); (2) Integration of gene annotations from Ensembl and miRBase [35] within secondary structure graphs (i.e., different genomic features such as exon, CDS, and 5’/3’-UTR regions are labeled with different colors); (3) Within the secondary structure graphs, users can search for specific linear motifs using regular expressions, and extract the structure graphs of sub-sequences; (4) In JBrowse page, categorized transcript tracks (i.e., protein-coding, miRNA, tRNA, rRNA, snRNA, snoRNA, transposable element, other ncRNAs, and miRBase primary miRNA transcript) are provided, and users can simply click a transcript in the JBrowse page and go to the corresponding structure viewer page; (5) In BrowserGenome view, exon densities and gene locations of different gene categories (i.e., protein-coding, miRNA, tRNA, rRNA, snRNA, snoRNA, transposable element, and other ncRNAs) can be clearly visualized. (6) Interactive search box (search-as-you-type) in the navigation bar enables users to find gene or transcript sequences annotated by TAIR10.27 (EnsemblPlants) or miRBase (Release 21) by an identifier, name or other keywords quickly.

### Function demonstration

#### Demo case of RNA secondary structure prediction

**Fig 1:**
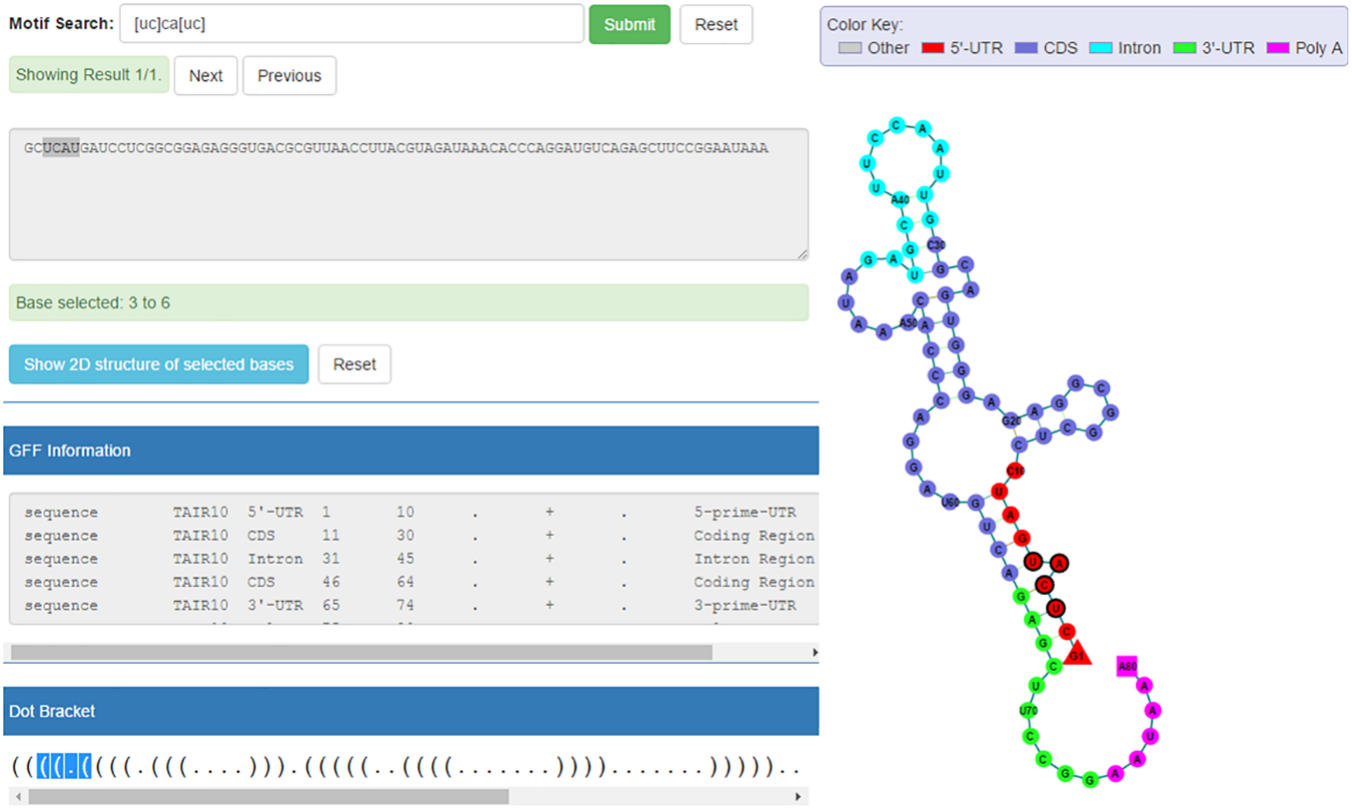
Web interfaces of RNA secondary structure prediction results. The web page shows the nucleotide sequence, genic annotation, DBN data, and secondary structure graph. In particular, the sequence segments with different annotation features (i.e., 5’ UTR, CDS, intron, 3’ UTR, etc.) are labeled with different colors. For demonstration purpose, we used the motif search function to search for the motif “[UC]CA[UC]” in this sequence, and found 1 match, which is highlighted in the nucleotide sequence, DBN data, and secondary structure graph correspondingly.

We predicted the secondary structure of a query RNA sequence (GCUCAAGAUCCUCGGCGGAGAGGGUGACGCGUUAACCUUACGUAGAUAAACACCCAGGAUGUCAGAGCUUCCGGAAUAAA) using RNAfold with default parameters and obtained its secondary structure graph on the web page (see Fig 1). The web page also shows the nucleotide sequence, genic annotation, DBN data, and secondary structure graph. In particular, the sequence segments with different annotation features (i.e., 5/3'-UTR, Intron, CDS, and Poly A site) are labeled with different colors. This is a novel, useful feature or function for biologists because any biological annotation (e.g., protein-binding sites annotation) can be integrated into the relevant secondary structures for exploring the connection of secondary structures and biological functions. For demonstration purpose, we used the motif search function to search for the motif “YCAY” (Y indicates pyrimidine, U or C), which is the binding site of neuronal-specific RNA-binding protein NOVA1 [36] using regular expression “[uc]ca[uc]”, and found 1 match inside a stem, with the matched result highlighted in the nucleotide sequence, DBN data, and secondary structure graph.

#### Selected Arabidopsis RNA secondary structures

**Fig 2:**
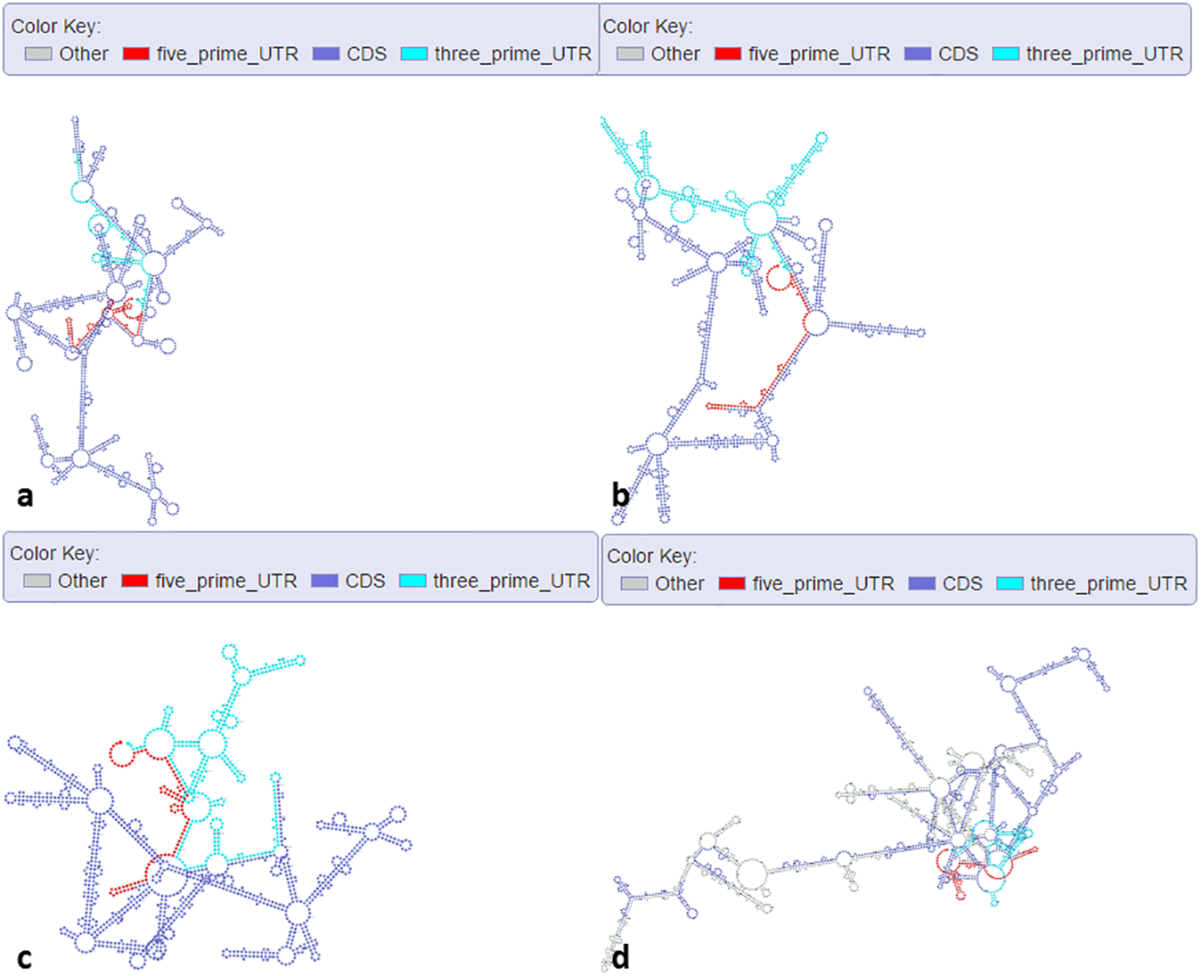
Secondary structure graphs of selected mRNA isoforms and un-spliced mRNA in *Arabidopsis*. (a) mRNA (Ensembl ID “AT4G27800.1”). (b) mRNA (Ensembl ID “AT4G27800.2”). (c) mRNA (Ensembl ID “AT4G27800.3”). D: un-spliced mRNA (Ensembl ID “AT4G27800.1”).

**Fig 3:**
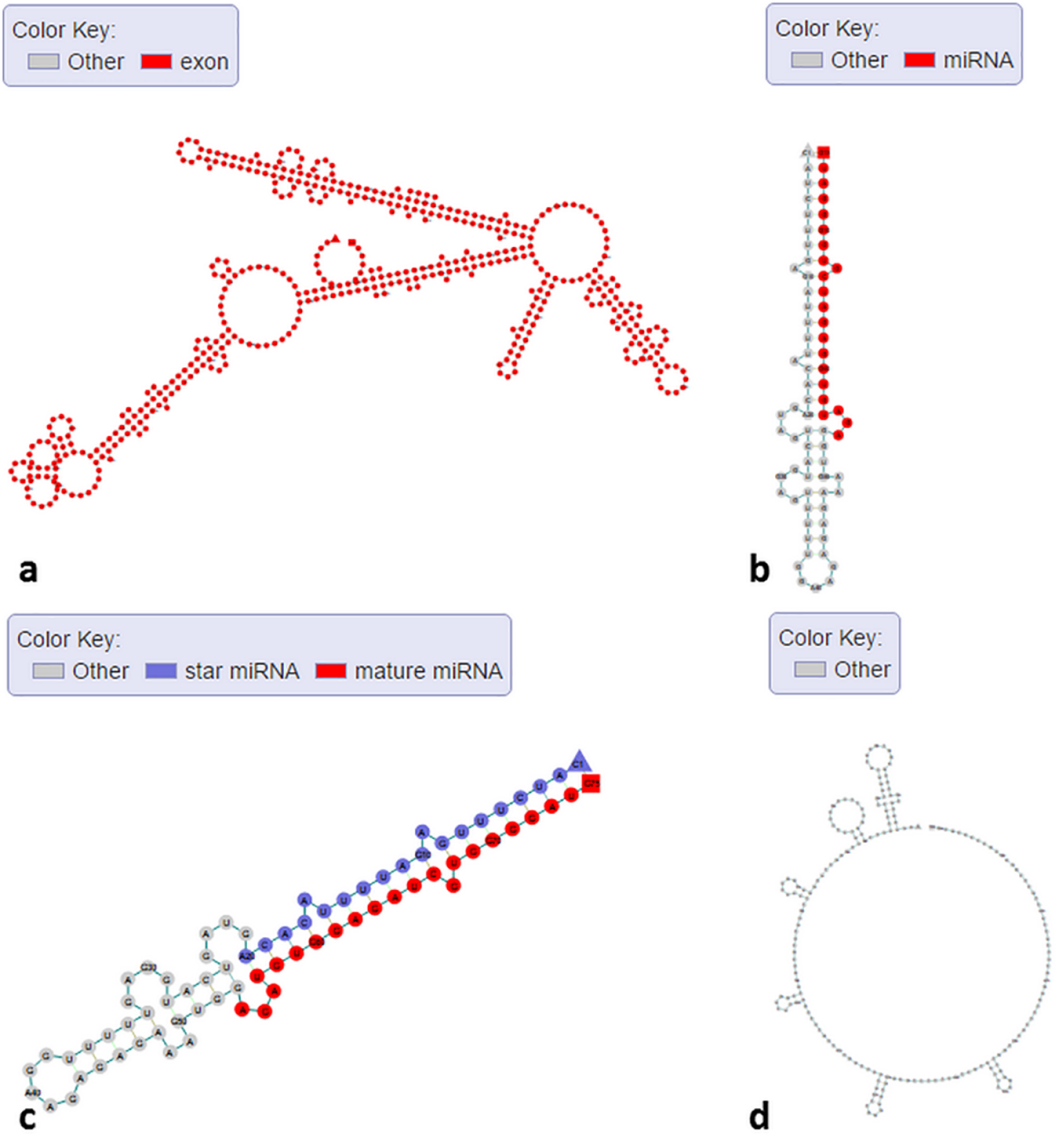
Secondary structure graphs of selected ncRNAs and ssDNA in *Arabidopsis*. (a) lncRNA (Ensembl ID “AT1G04425.1”). (b) miRNA (miRBase ID “MI0031741”). (c) miRNA (miRBase ID “MI0031741”) with customized annotation information (star miRNA position). (d) ssDNA (chromosome: 1; start: 200; end: 400; strand: +).

**Fig 4:**
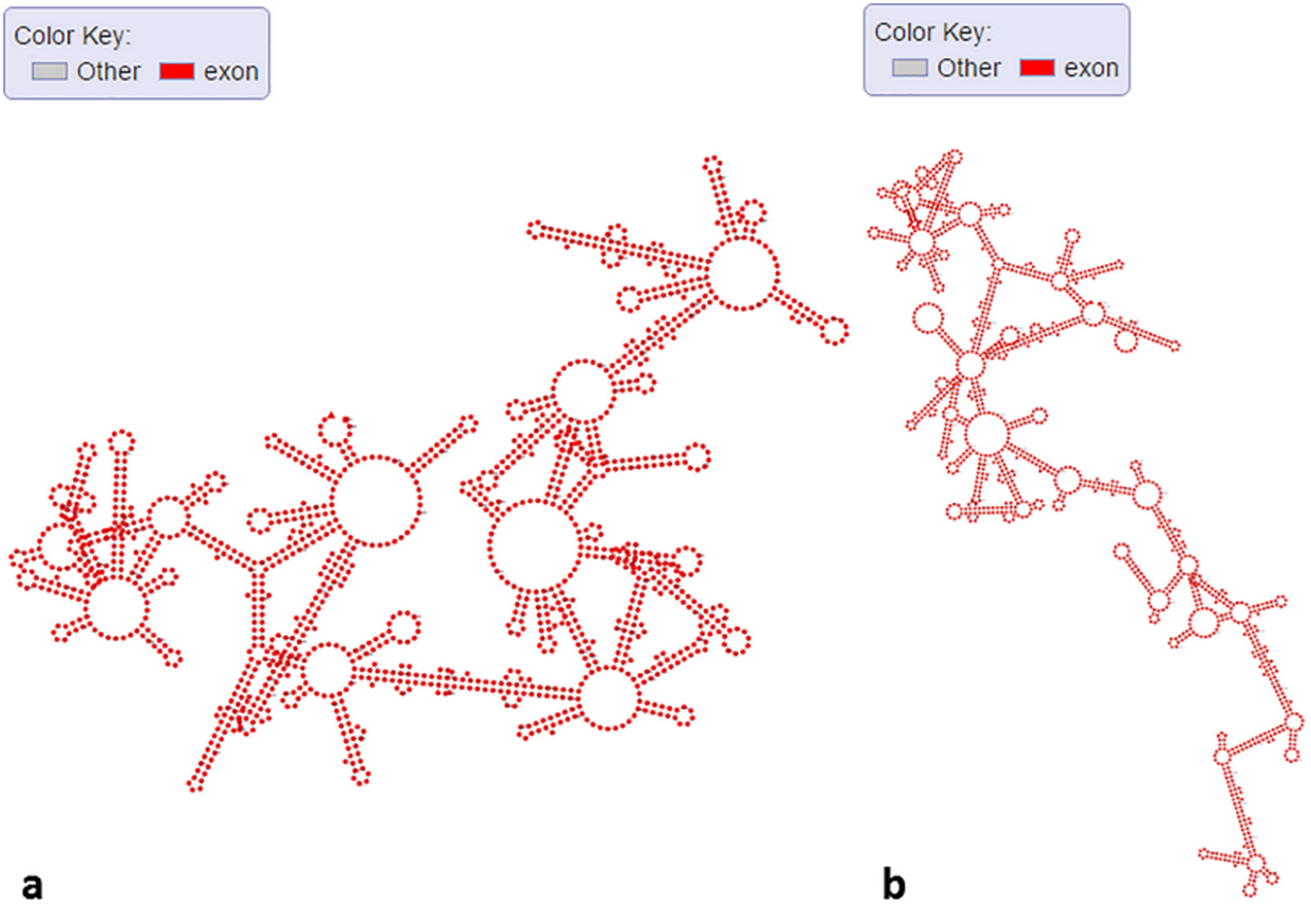
Secondary structure graphs of a selected rRNA in *Arabidopsis*. (a) rRNA (Ensembl ID “ATCG00920.1”) without experimental data as constraints. (b) rRNA (Ensembl ID “ATCG00920.1”) with experimental data as constraints.

We used RNAfold with default settings to predict RNA secondary structures of several transcripts of *Arabidopsis* and showed their structure graphs. In Fig 2A–2C, we showed the structures of different mRNA isoforms (Ensembl ID “AT4G27800.1”, “AT4G27800.2”, and “AT4G27800.3”). These isoforms are transcribed from gene “PPH1” (Ensembl ID “AT4G27800”), which codes for “Protein phosphatase 2C 57”. It is clearly that these isoforms show different secondary structures, which might be related to their translation activity. In Fig 2D, we showed the structure of one un-spliced mRNA (Ensembl ID AT4G27800.1) that contains introns. The 5'-UTR, 3'-UTR, and CDS regions of the transcripts are labeled with different colors, and the introns are labeled as “Other” in gray color. Users can visualize and compare the structure features between spliced and un-spliced RNAs to understand the discrepancy and explore the connection between different genic features and secondary structures. In Fig 3A, one lncRNA (Ensembl ID “AT1G04425.1”, length>200 nt) generated from different exons of its gene displays a complicated secondary structure. In Fig 3B, a miRNA (miRBase ID “MI0031741”) shows the typical hairpin structure with the mature miRNA arm highlighted in red in this primary miRNA transcript. After we uploaded this sequence with additional annotation information (star miRNA position), mature miRNA (red), star miRNA (dark blue), and loop (gray) can be clearly separated (see Fig 3C). The capability of our web interface in displaying differential color schemes for different sequence features or genic annotations can facilitate data visualization and validation. We randomly selected a region (chromosome: 1; start: 200; end: 400; strand: +) of *Arabidopsis* genome and used Mfold with default parameters to predict the ssDNA secondary structure of this region. Fig 3D shows that some parts of the ssDNA can form the palindrome structures (inverted repeats), which are involved in many biological processes including DNA replication [37], DNA transition [38] and DNA methylation [39]. Users can use our web server to view the ssDNA structure of their interested regions in *Arabidopsis* genome. We also used StructureFold with default parameters (RNAstructure as prediction module) to predict the *in vivo* RNA structure of an rRNA (Ensembl ID “ATCG00920.1”) with experiment data as constraints and compared it with the in silico RNA structure (RNAfold with default settings) (see Fig 4A and 4B). rRNA structures are crucial in the process of translation [40–42], and relevant structure study can help understand the mechanism of translation. It is clear that those two structures are quite different. Without the *in vivo* experimental data as computational constraints, it’s hard to get the RNA structure accurately, because the intracellular environment is complicated and many factors such as proteins can bind RNAs to change their naturally folded structures [43–45]. Users can use StructureFold with customized settings to predict RNA structures incorporating *in vivo* profiling data and visualize them in our server.

#### BrowserGenome and JBrowse integration

**Fig 5:**
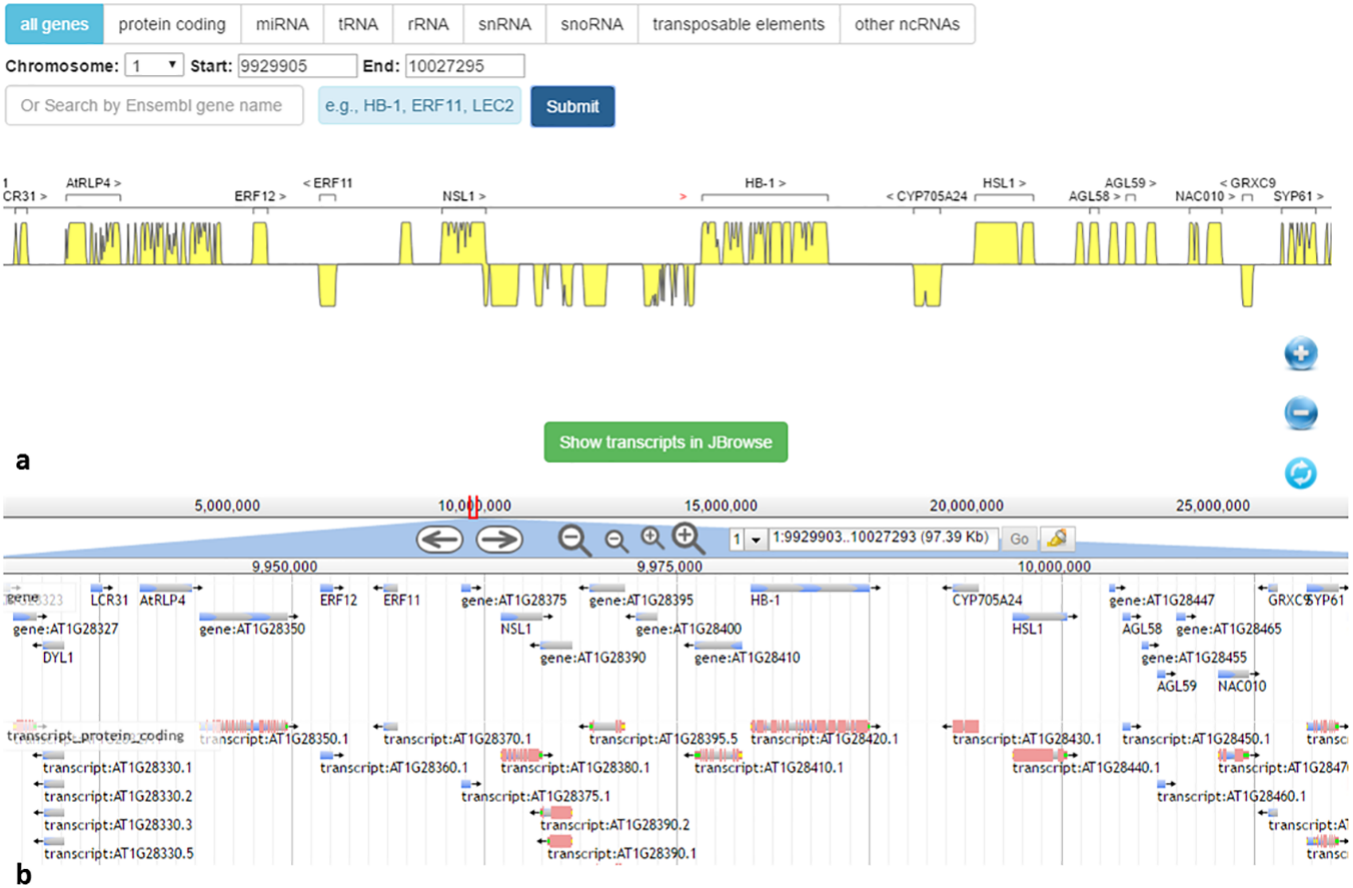
Genome browser views of a randomly selected region in Arabidopsis thaliana genome. (a) BrowserGenome view of the selected region (chromosome: 1; start: 9929905; end: 10027295). (b) JBrowse view of the selected region (chromosome: 1; start: 9929903; end: 10027293).

BrowserGenome provides a bird-eye density view for the reference genome, while JBrwose provides a very detailed view of genes and their isoforms, so we need both of them to visualize all the useful genic information. In BrowserGenome, we randomly selected a region (chromosome: 1; start: 9929905; end: 10027295) of *Arabidopsis* genome in BrowserGenome (see Fig 5A), and clicked the “all genes” tab. The exon densities and gene locations for “all genes” can be clearly visualized. Users can zoom in the density graph to a resolution where individual gene names are shown up, with a button that can lead to JBrowse view of detailed gene and isoform structures. Users can also click other tabs to view different categories of genes, search for specific genes, zoom in/out, or show transcript in JBrowse view. In JBrowse, we selected a similar region (chromosome: 1; start: 9929903; end: 10027293) of *Arabidopsis* genome (see Fig 5B), and enabled the gene and protein-coding transcript tracks. We labeled different genomic features with different colors (e.g., CDS: pink; 5'-UTR: yellow; 3'-UTR: green), making it easy for users to separate the annotation features. Users can click a transcript on the track, and go to the corresponding structure viewer panel, while clicking on a gene in the track will pop up a window that contains sequence information.

## Discussion

For a given sequence in FASTA format, we have provided 5 popular programs (RNAfold, RNAshapes, RNAsubopt, RNAstructure, and Mfold) to conduct RNA secondary structure prediction. Among them, RNAstructure and Mfold can be also used for DNA secondary structure prediction [20,21,46–48]. Users can customize the prediction algorithm and set some advanced parameters in each of these programs on our web server. This design enables biologists to perform comparative secondary structure analysis with different prediction programs/algorithms, or even with different parameters within the same program, which is important because: (1) no structure prediction algorithm is the best or superior one, always with tradeoff and pros and cons, and (2) consensus approaches are widely used in bioinformatics. For example, Maker [49] uses such consensus approaches in gene prediction. In particular, gene annotations keep improving for many species including model species like *Arabidopsis*, as more and more data are being generated. Therefore, it is highly likely that new transcript units can be found in the genome, in addition to the current annotation. So, it is necessary to examine any region in the genome to study its ssDNA structures and secondary structures of relevant putative RNA transcripts. Accordingly, our web portal allows biologists to choose a specific ssDNA segment of *Arabidopsis* genome and predict its secondary structure and relevant RNA secondary structure. If users want to be more flexible about the prediction algorithm, they can run different algorithms locally on their own computers or in the official web servers of the aforementioned 5 tools, and then upload the secondary structure result files in DBN or CT format to our server to view the structure graphs. In addition, users can add customized genomic annotations in GFF format to the structure graphs, in order to study the structural differences among different annotation features. Users can also search for any interested linear sequence motif (minimum length = 3) using a regular expression and highlight them within the structure graphs. This feature enables biologists to detect the secondary structure of specific motifs within the query sequence. We noticed that our 2D layout algorithm needs to be improved because some structure motifs are overlapped to some extent when the input query sequence is very long. Similar problems occur in other popular tools like Mfold [21], RNAfold [25] and RNAshapes [31]. However, we allow users to specifically extract a part of the structure graph and view the sub-structures in a new panel when the whole structure graph is complicated and overlapped. Moreover, our structure viewer provides accurate and interactive correspondences between sequence nucleotides, structure graphs, Dot-Bracket data, and GFF features, which are not available in any of other aforementioned tools.

As a powerful DNA/RNA structure visualization tool, JNSViewer is very important for constructing computational molecule predictors, such as miRNA predictors based on RNA secondary structures. Recent useful miRNA prediction tools such as iMiRNA-PseDPC [50], miRNA-dis [51], and iMcRNA-PseSSC/iMcRNA-ExPseSSC [52] are all based on RNA secondary structures. These miRNA predictors extract feature vectors based on the structure-order information of RNA sequences and use machine learning algorithms to accurately predict pre-miRNAs, and they are useful high-throughput tools for genome analysis with large-scale data. Experimental data can improve the accuracy of computational prediction of RNA secondary structure. Recently, several studies [4,53–55] have incorporated the experimental data from high-throughput sequencing in computational structure prediction, and have new discoveries in the connection between gene expression regulations and RNA secondary structures. We utilized the StructureFold [29], an open-source and generic pipeline in analyzing high-throughput structural sequencing data and predicting RNA secondary structures with *in vivo* experimental evidence from RNA structure profiling data as constraints. Our prediction results might be a useful resource for *Arabidopsis* RNA structure and function research, because we provided interactive 2D graph views of putative *in vivo* RNA structures supported by experimental data, which are not available on other web servers. Our Linux server stores the sequence FASTA files, annotation GFF files, and structure DBN and SSDJ files for individual transcripts, and the Linux file system can provide users a quick access to the data. Using our web server, biologists can easily compare the secondary structures of different mRNA isoforms with the combination of bundled genome browsers (JBrowse and BrowserGenome) and structure viewer. In particular, for miRNAs, we downloaded useful annotation information, including the positions of mature miRNA arms in the precursor sequences, from miRBase and integrated it in both JBrowse and structure viewer page, providing users detailed structure information for miRNAs for visual validation.

We created an easy-to-read, easy-to-use and lightweight data format called secondary structure dataset in JSON (SSDJ) that not only stores base-pairing information for DNA/RNA secondary structure as in DBN and CT formats, but also contains graphic drawing information like 2D coordinates and color setting for each base. The light-weight nature of SSDJ enhances the execution speed in drawing secondary structures and efficiency of data retrieval and communication over the Internet. Moreover, different from DBN and CT formats, SSDJ is an extendable format based on JSON, which can be effectively utilized and integrated into web-based programming, and can also be implemented using different computer programming languages such as C++, Java, JavaScript, Python, and so on. Our web server has separate steps for 2D graphic layout (i.e., assigning graphic information to each base) and secondary structure drawing. This is very different from existing software for RNA secondary structure prediction [21,25,31,47,56,57], where the steps of 2D graphic layout and drawing are bundled together. The advantage of our approach is to facilitate the modularity and interoperability among different 2D graph layout and drawing tools. Better graph layout programs can be integrated into our web server easily. In addition to our SSDJ format, popular RNA/DNA structure formats such as DBN and CT can be efficiently utilized in our web server.

## Conclusions

For biologists, JNSViewer offers a user-friendly web interface that can present precise connection among nucleotide sequences, pairing and folding information, biological annotations, and secondary structure graphs. The useful features such as annotation integration, motif search and highlighting, comparative visualization of secondary structures, and genome browser integration will empower biologists in plant RNA study.

## Methods

### Web interface and secondary structure prediction

To develop the web interfaces, we have utilized the CSS library: Bootstrap (http://getbootstrap.com/; version 3.3.6) and JavaScript libraries: jQuery (http://jquery.com/, version 1.12.0) and Angular JS (https://angularjs.org/; version 1.5.2). We used JavaScript to create SVG images for both nucleotide secondary structure graphs and dot-bracket notation data, as well as an HTML-based text field of nucleotide sequences dynamically. We also offered some useful client-side JavaScript functions, such as nucleotide position ruler, annotation file integration, linear sequence motif search using regular expressions, structure motif highlighting, and sub-sequence structure graph extraction, which are very generic and browser independent. The web interface is well tested in Mozilla Firefox (version 44+) and Google Chrome (version 45+). The server-side data processing pipeline is implemented by PHP (see S1 Fig), which invokes both third-party and our own C++ and Perl programs. The third-party programs include RNAfold (version 2.2.5), RNAshapes (version 3.3.0), RNAsubopt (version 2.2.5), RNAstructure (version 5.7) and Mfold (version 3.6). All these programs can be used for RNA secondary structure prediction, and RNAstructure and Mfold can be used for ssDNA secondary structure prediction. We have mainly developed the following C++ programs on the server side: (1) “*cttodbn*” is used to convert an input data file in CT format into DBN format; (2) “*dbtoss*” is a program that takes DBN data input, utilizes our own 2D graphic layout algorithm and generates SSDJ data output; (3) Using GD Graphics Library (http://libgd.bitbucket.org), “*ssdjtopng*” is for importing input data in SSDJ format and creating high-quality PNG images for download; and (4) “*ssdjtosvg*” is a program that takes SSDJ input data and generates SVG images for download.

### SSDJ definition and specification

SSDJ is an easy-to-use and light-weight data format designed by us for effective exchange of critical information in RNA secondary structure visualization. An XML-based data format known as RNAML had been created previously for exchanging basic RNA molecular information [58]. Unfortunately, RNAML did not gain any popularity in its usage due to its complexity and heavy-weighted nature. Based on JSON, our SSDJ format only stores critical base-paring and drawing information for RNA secondary structures. As shown in S2 Fig, SSDJ primarily consists of 5 parts.

1. sequence: the sequence bases (nucleotides) in the secondary structure graph.
2. dot bracket: the dot bracket notation that shows base-paring and folding information.
3. coordinate: the two-dimensional positions or coordinates of every base in the secondary structure graph. Each base is positioned at the coordinates x and y. One space or semicolon is treated as the separator between coordinates.
4. color: this part of information is optional, which will provide coloring information for each base in the secondary structure graph. A group of valid 6-digit hex number (from 000000 to FFFFFF) with a prefix of # is used to define the color, and a space or a semicolon is used as the separator for each group (each base). If users do not provide the color information, a color schema based on different nucleotides (i.e., four different nucleotides are assigned with different colors) will be utilized as the default. In RNAfold web server (http://rna.tbi.univie.ac.at/cgi-bin/RNAfold.cgi), the color has been utilized to represent either base-pair probabilities or positional entropy. SSDJ provides a possibility for such integration as long as an SSDJ file with proper color assignment is provided.
5. pairings: the indexes of the base pairs. The index of each base starts at 1 and increases by 1. A group of indexes *n*, *m* means that the base *n* is paired with the base *m*. One space or one semicolon is used as the separator to identify each pair.

### 2D graphic layout and drawing

Different algorithms in RNA secondary structure drawing have been proposed [25,59,60]. Usually, the 2D graphic layout for each nucleotide and actual 2D drawing are bundled as in popular tools like Mfold, RNAfold, and RNAshapes. Differently, we have separated these two steps (i.e., “*dbtoss*” for 2D graphic layout and “*ssdjtopng*” or “*ssdjtosvg*” for 2D drawing) for modularity and incorporation of SSDJ that will enhance extensibility, comparability, and compatibility among different tools and algorithms in RNA secondary structure prediction and visualization. In our 2D graphic layout algorithm, nucleotide bases are grouped according to base-pairing information presented in DBN format. Using the same definitions adopted previously [59], groups are classified into structure motifs including S = stem, B = bulge, H = hairpin, I = interior loop and M = multi-branch loop. According to this classification, each base within a given group (i.e., S, B, H, I or M) is assigned with proper 2D coordinates with color information, resulting in a raw graphic dataset for the given group that represents a subgraph in the final structure graph. In order to form the final graphic dataset, the raw coordinates of each subgraph must be transformed to the final coordinates so that subgraphs are smoothly combined to form the final secondary structure graph with an appropriate 2D layout where the overlaps among subgraphs are minimized. We noticed that our 2D layout algorithm needs to be improved because some structure motifs are overlapped to some extent when the input query sequence is very long. Similar problems occur in other popular tools like Mfold, RNAfold, and RNAshapes. What is important here is the modularity that we brought using SSDJ, which makes it possible that different research groups can focus on 2D graphic layout algorithms to improve RNA drawing tools in the future. In addition to SSDJ format, popular RNA/DNA structure formats such as DBN and CT can be efficiently utilized in our web server.

### *Arabidopsis* RNA secondary structure prediction

We downloaded the genome sequences and gene annotation (GFF3) of *Arabidopsis thaliana* (TAIR10.27) from EnsemblPlants website (http://plants.ensembl.org/index.html), and used our own Perl scripts to extract individual sequences and annotations for all coding and non-coding RNAs in both spliced and unspliced forms (e.g., mature mRNAs with their major isoforms vs their pre-mRNAs). We used RNAfold with default parameters to predict the secondary structures in DBN format for all transcripts and transformed the DBN files to SSDJ files for structure 2D presentation. The sequences, genic annotations and structure files for individual transcripts were stored in separate folders on our web server, and users can quickly view the structure graph of the query RNA sequence by entering the Ensembl ID. Particularly, for miRNAs, we also downloaded useful annotations from miRBase (release 21), used Perl scripts to extract the annotations including mature arm positions for primary miRNA transcripts, and predicted the secondary structures using RNAfold with default parameters, which can be accessed by miRBase ID. We chose RNAfold because it is the most popular RNA secondary structure prediction tool [30,56,61], and has the highest speed among 5 tools. In addition, we also allow users to customize the prediction algorithms for the query sequences of *Arabidopsis*, or choose a specific segment of the genome DNA sequences to predict the structure of ssDNA or putative transcribed RNA. For the customized prediction of *Arabidopsis* DNA or RNA structure, the process will be on the fly.

To incorporate the *in vivo* experimental data in RNA secondary structure prediction, we first downloaded the sequencing data of previous RNA structure study [4] in FASTQ format from NCBI SRA database (http://www.ncbi.nlm.nih.gov/sra) with accession number “SRR933551”, “SRR933552”, “SRR933556” and ‘‘ SRR933557”. Then we utilized StructureFold (Repository version 119, https://toolshed.g2.bx.psu.edu/repository?repository_id=00fdabcadd09fb14&changeset_revision=7bb98e9296e9) pipeline with default settings and RNAstructure as the prediction module to incorporate the above sequencing data as experimental evidence and predict the secondary structures of all transcripts (coding and non-coding RNAs) of *Arabidopsis* (methods see S1 File). The StructureFold output results in CT format were then converted to DBN format, and then converted to SSDJ format. Our server stores two versions of *Arabidopsis* RNA secondary structures (with and without the integration of *in vivo* RNA structure profiling data) for each transcript. Users can compare the two versions of structures to find the differences between *in vivo* and *in silico* RNA structures. For some transcripts, the version with *in vivo* experimental data is not available because the RNA profiling experimental data is not available for these transcripts.

### Genome browser integration

For BrowserGenome (http://www.browsergenome.org/), we downloaded its source codes and modified/customized the HTML, CSS, and JavaScript files to build our own customized version for *Arabidopsis*, and integrate the customized genome browser in an iframe in JNSViewer index page. In particular, we created views for different categories of genes (i.e., all genes, protein-coding, miRNA, tRNA, rRNA, snRNA, snoRNA, transposable element and other ncRNAs), so that users can easily browse and navigate different categories of RNAs. When users zoom into a certain resolution level, a button is available to let users examine the detailed gene and transcript isoform structures in JBrowse.

For JBrowse (http://jbrowse.org/), we installed version 1.11.6 on the server, and built the reference genome track and individual transcript tracks (i.e., protein-coding, miRNA, tRNA, rRNA, snRNA (small nuclear RNA), snoRNA (small nucleolar RNAs), transposable element, other ncRNAs, and miRBase primary miRNA transcript). We customized the CSS style of JBrowse to show 5’-UTR (yellow) and 3’-UTR (green) region in different colors, and added own JavaScript codes to implement the following functions: (1) redirecting to the corresponding structure viewer page after users click an RNA sequence in the transcript track in JBrowse; (2) adding a search box in JBrowse for users to search for gene or transcript by gene ID (Ensembl or miRBase) or name. We also added a JBrowse link in the structure viewer result page for each query *Arabidopsis* transcript, which will redirect users to the correct genome location in JBrowse with the query sequence highlighted in yellow.

### File system

JNSviewer adopts project concept for data processing and file storage. When a user uploads data in FASTA or other formats, a project with a specific date and time stamp will be created, which mirrors a specific folder on our web server that hosts all data relevant to that project. There is no limitation on how many projects a user can create. Every user's data is private and protected by a randomly generated access code and a project name that contains a randomly generated unique code with a date and time stamp. The user ID and access code to access the project data will be provided at the time of data submission. All the data will be kept on our server for a week and will be removed automatically afterward. Before removal, all projects and associated data can be recovered using the access code.

## Supporting information

S1 Fig Data processing pipeline and flowchart in JNSViewer. (TIF)

S2 Fig Definition of RNA secondary structure dataset in JSON (SSDJ). (TIF)

S1 File Methods for RNA secondary structure prediction with StructureFold. (DOCX)
