# Supplementary figures and images for: JNSViewer—A JavaScript-based Nucleotide Sequence Viewer for DNA/RNA secondary structures
Source: PLoS One. 2017 Jun 5;12(6):e0179040. doi: 10.1371/journal.pone.0179040 (PMC5459502; doi:10.1371/journal.pone.0179040)

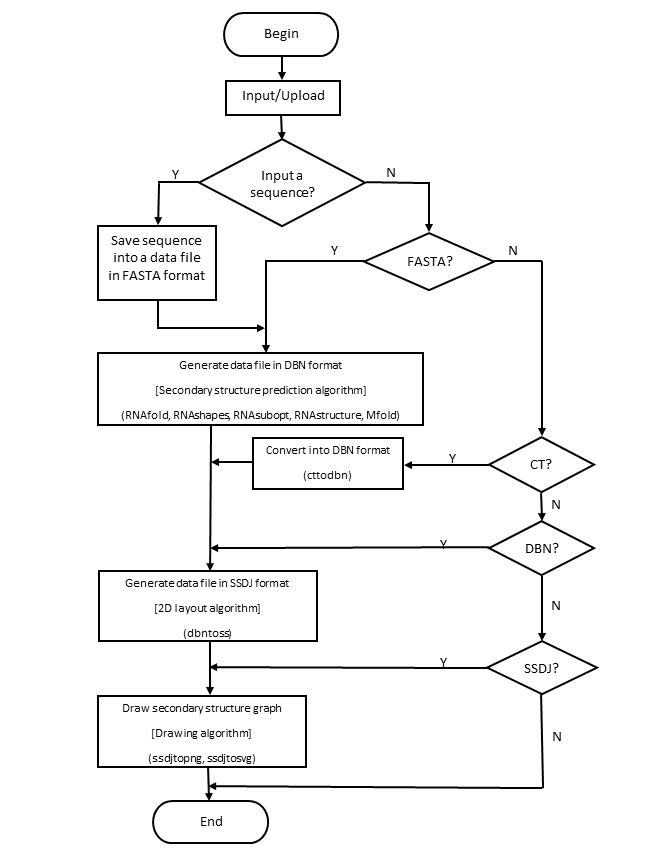

Supplement: S1 Fig — (TIF) [file pone.0179040.s001.tif]

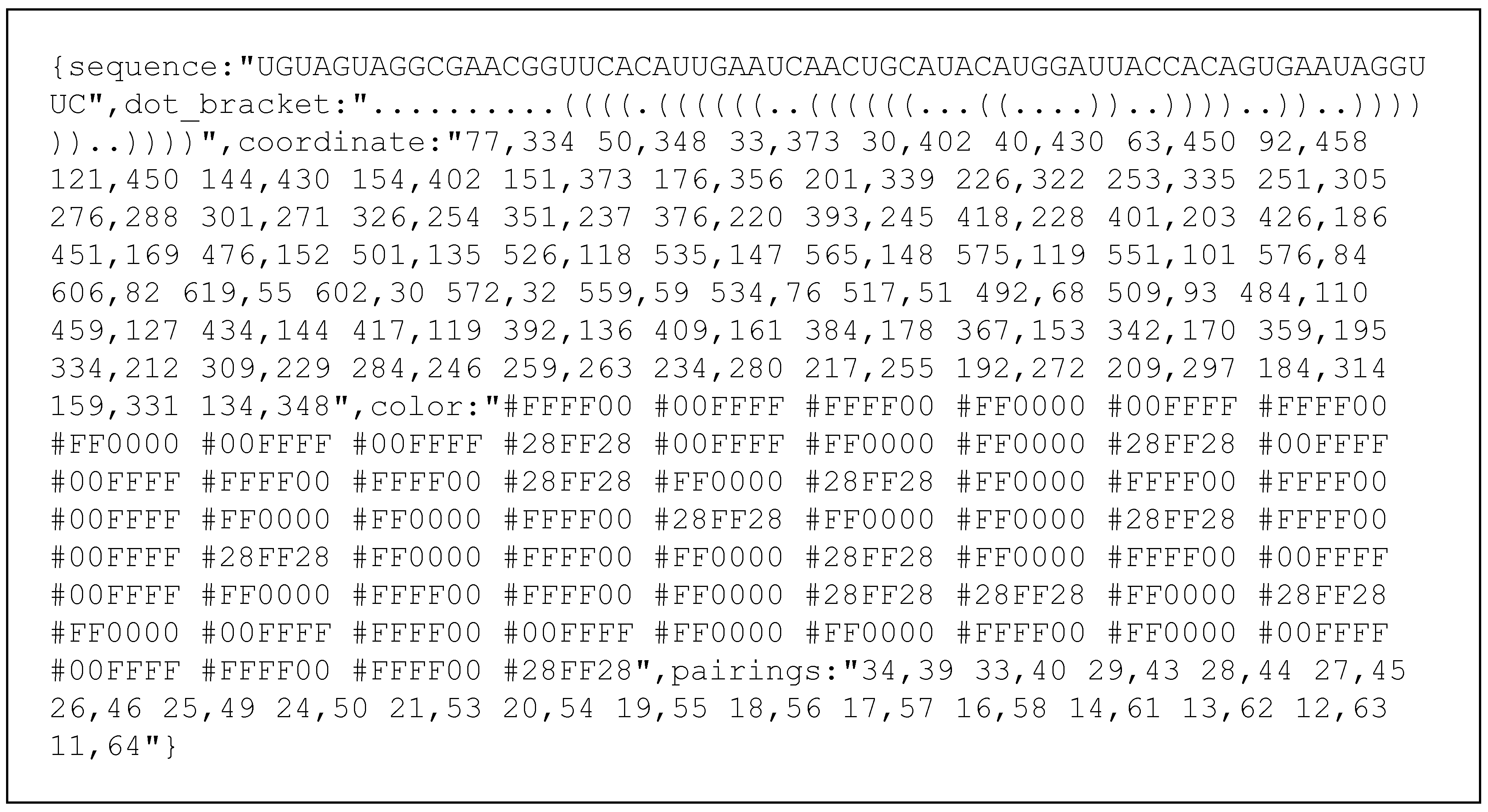

Supplement: S2 Fig — (TIF) [file pone.0179040.s002.tif]
