# Supplementary material for: JNSViewer—A JavaScript-based Nucleotide Sequence Viewer for DNA/RNA secondary structures
Source: PLoS One. 2017 Jun 5;12(6):e0179040. doi: 10.1371/journal.pone.0179040 (PMC5459502; doi:10.1371/journal.pone.0179040)
Supplement: S1 File — (DOCX) [file pone.0179040.s003.docx]

**Methods for RNA secondary structure prediction with StructureFold**

Our data analysis uses the data mentioned in Ding’s paper (<http://www.nature.com/nature/journal/v505/n7485/full/nature12756.html>), and uses the software package provided in StructureFold paper (<http://bioinformatics.oxfordjournals.org/content/31/16/2668.long>). The detailed steps are as follows.

(1) Download sequencing data “SRP027216” (4 FASTQ files) from NCBI SRA database.

(2) Download StructureFold package from Galaxy Tool Shed: <https://toolshed.g2.bx.psu.edu/repository?repository_id=00fdabcadd09fb14&changeset_revision=7bb98e9296e9> (version 119 (2015-04-14)).

(3) Download the “cdna” and “ncrna” sequences of Arabidopsis (TAIR 10 release 27) from Ensembl Plants database (<ftp://ftp.ensemblgenomes.org/pub/plants/release-27/fasta/arabidopsis_thaliana>).

(4) Use “structurefold/Iterative_mapping/iterative_map.py” for iterative mapping of the 4 input FASTQ files. The command is “python iterative_map.py fastq <input_FASTQ> <reference> 1 21 3 default <output_BAM>”. Here, <reference> is downloaded “cdna” or “ncrna” sequence file.

(5) Combine the output BAM files of “iterateive_map.py” from the same library type (+ or -) to one BAM file, and sort the combined BAM files. Originally, we have 4 BAM files (two for +, and two for -), and now we only have 2 combined BAM files (one for +, and one for -).

(6) Use “structurefold/get_reads/get_read.py” to get RT stop counts from the BAM files. The command is “python get_read.py <input_BAM> <output_count>”. Here, we have two input BAM files (+/-), so there are two output count files (+/-).

(7) Use “structurefold/reactivity_cal/react_cal.py” to calculate reactivity. The command is “python react_cal.py <plus_count> <minus_count> <reference> AC 1 7 <output_reactivity>”. Then select the ids of sequences that have reactivity in the output reactivity file. The ids are stored in a file.

(8) Use “structurefold/predict/predict_RNAs.py” to predict RNA structure. The command is “python predict_RNAs.py <intput_id_file> <reference> restraint 310.15 rs <output_html> <output_dir> <reactivity_file> 1.8 -0.6”. Here, we removed the limit of the number of sequence to be predicted (N=100) in “predict_RNAs.py”.

(9) Covert output results in CT format to DBN format.
